# Background, establishment and initial experiences of the Danish cardiovascular homograft biobank

**DOI:** 10.1007/s10561-024-10137-0

**Published:** 2024-07-27

**Authors:** Margrete Stenehjem, Dorte Kinggaard Holm, Lars Riber, Christian Nielsen, Sara Schødt Riber, Cengiz Akgül, Jes S. Lindholt

**Affiliations:** 1 Department of Cardiothoracic and Vascular Surgery T, Odense University Hospital, J.B. Winsløws Vej 4, 5000 Odense C, Denmark; 2 Clinical Immunology Research Unit, Department of Clinical Immunology, Odense University Hospital, Odense C, Denmark; 3 Department of Clinical Research, University of Southern Denmark, Odense C, Denmark

**Keywords:** Vascular homograft, Vascular allograft, Biobank

## Abstract

Odense University Hospital is a major tertiary vascular hospital in Scandinavia, performing approx. 200 aortic repairs annually. This article presents the rationale behind this endeavor and the early outcomes of the initial implantation of locally processed homografts. All patients receiving a homograft were identified from the established homograft biobank database and their medical records were reviewed after obtaining consent. All surgeons in charge of homograft implantations were semi structured interviewed regarding the harvesting procedure, the tools for detecting available homografts, their quality and delivery. The National board of Health approved the biobank fulling the EU Directive of Tissues and Cells after 18 months of preparation. From May 6, 2021, to March 1, 2023, 26 patients had a homograft implantation, with 7 for mycotic aneurysms, 10 for aorto-iliac graft infection, 6 for infra-inguinal graft infection, and 3 for graft infection in thoracic aorta. Six (23%) were emergently performed. Two (7.7%) died within 30 days postoperatively, both following in situ replacement of an infected aortoiliac graft, corresponding to a 20% mortality in this subgroup. The incidence of reinfections was 19.2%; one each in the mycotic aneurysm group, the aortoiliac graft infection group, and the thoracic graft infection group. After 90 days, two patients were diagnosed with aorto-enteric fistula. All involved surgeons could easily identify available suitable homografts, and within 2 h have homografts of acceptable quality and requested dimensions. The establishment of the Danish Cardiovascular Homograft Biobank was straightforward and effectively serves cardiovascular procedures performed 24/7. Additionally, the initial experiences seem comparable to others experiences.

## Introduction

In 2019, vascular and heart surgeons, together with clinical immunologists at Odense University Hospital (OUH), collaborated to establish Scandinavia’s first cardiovascular homograft biobank. Here we provide the rationale behind this endeavor, and the process involved in establishing the biobank and the first-year experiences encountered during the 2 years of using the tissue bank.

### Definition

A cardiovascular homograft (allograft) is a transplanted heart valve or blood vessel from another person.

### History

The first human uses of cardiovascular homografts were described in 1906 (Guevara-Noriega et al. 2019), but their wide adoption began with Fontaine and Leriche´s establishment of the first homograft tissue bank for clinical use in 1951. Dubost immediately took advantage of this innovation, executing the historical first abdominal aortic aneurysm (AAA) resection successfully, which attracted global attention. Prominent vascular surgeons like DeBakey and Szilagyi quickly adopted the procedure, leading to the establishment of biobanks for frozen homografts. In 1952 a patient in New York presented with a ruptured AAA, but the homograft biobank was empty. Dr Voorhees improvised by making a tube graft from a World War II parachute, achieving a successful implantation. The procedure was then offered to e.g. Nobel laureate Albert Einstein, who declined, and died in 1955 from a ruptured AAA (Friedman 2005). Subsequently, the industry started production of artificial blood vessels, including those made from materials such as Dacron and Teflon-the latter as product development of the Apollo 11 astronaut suits. Later advancements led to the preference for artificial heart valves over homografts. This development also introduced a new and feared complication, prosthesis infection. Antibiotic treatment alone is insufficient to eradicate the infected surrounding biofilm, necessitating surgical treatment as the only curative option. This typically involves replacing the infected graft with a similar implant, which inherently carries increased risk of infection. Consequently, there was a resurgence in the use of homografts, especially in the USA, Central and Southern Europe, albeit limited to replacing infected arterial prostheses, complex arterial segments and lesions, or dialysis accesses (Guevara-Noriega et al. 2019; Wyburn 1959 Mar).

**Fig. 1:**
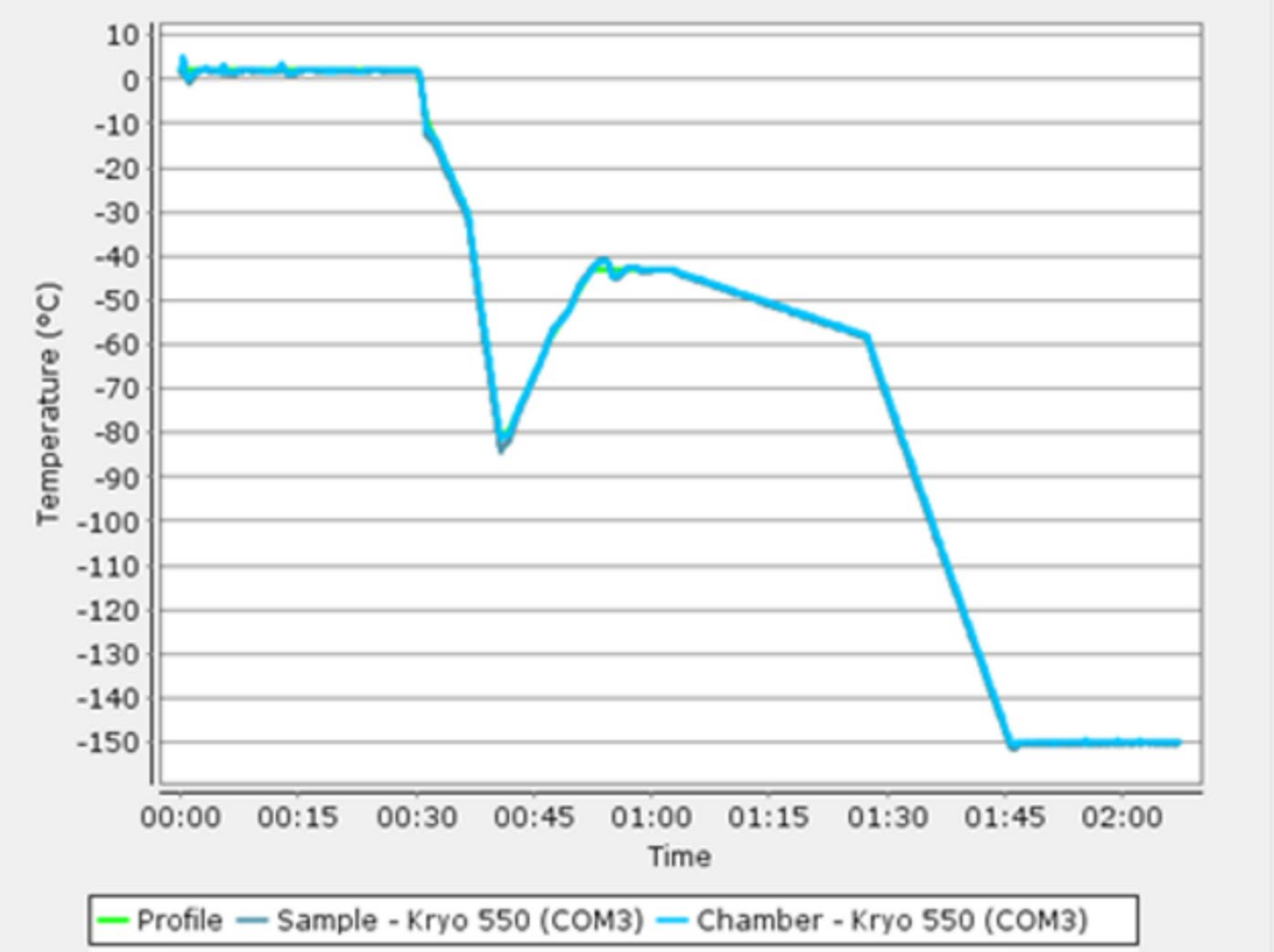
Example from the freezing temperature log of a homograft in Odense Biobank showing the differentiated cryofreezing preventing crystallization of the tissue together with anticrystallization agents

However, it became evident in the 50 s that frozen grafts quickly degenerated due to tissue crystallization during freezing. Towards the end of the millennium a rate-differentiated freezing method was discovered, utilizing liquid with anti-crystallization agents, which preserved cellular vitality (Fig. 1) and since then, the use of cardiovascular homografts have increased significantly with promising results (Chiesa et al. 1998; Bisdas et al. 2010; Yanagava et al. 2018) to the extent that they have also been used in bypass surgeries for both cardiac and peripheral vascular indications.

### Indications for use of homograft in our time, according to recent literature

There is now a broad spectrum of indications for use of homografts.

Considering larger meta-analyses (Yanagava et al. 2018; Williams et al. 2022) the Society of Thoracic surgeons recommend homograft utilization for patients with extensive active endocarditis with destruction of the aortic ring and it should be considered particularly in cases where the risk of reinfection is increased.

For aorto-iliac/aorto-femoral graft infections, conservative treatment with antibiotics will never be curative. In situ repair seem to give fewer complications and reinfections and lower mortality rate compared to extra anatomic bypass (Chafké et al. 2020; Antonopoulos et al. 2019 Jul; O´Connor et al. 2006; Batt et al. 2018; Niaz et al. 2020). Under those circumstances reconstruction by patients own veins is time consuming, a huge stress for the patient, and is associated with high amputation rate. Homografts do as well or better than synthetic grafts, and according to a recent multicenter study homograft in the aortoiliac/femoral segment have a primary patency of 97% after 5 years.

For mycotic aneurysms the use of homograft presents the opportunity to remove the infected arterial tissue, do debridement and in-situ reconstruction, if the patient is stable for such a large procedure.

For peripheral bypass use, V. Saphena Magna is reported to have a primary durability of 82% after 1 year, and 64% after 5 years, comparable to patency for in situ bypass (Randon et al. 2010; Buckley et al. 2017; Martin et al. 1994; Faber et al. 2003). Femoral arteries and deep veins could also be used.

Until now, we have limited the use of homograft for infrainguinal bypasses for those with infection.

Experienced with saphenous vein homograft for coronary bypass is modest (Laub et al. 1992).

A sixth indication for use of homograft is in complicated cases of arteriovenous access for dialysis, when the patient lacks usable vein, and infection constitutes a relative contraindication for synthetic bridge graft (Almasri et al. 2016; Lin et al. 2002; Matsuura et al. 2000, 2002) (https://www.cryolife.com/wp-content/uploads/stories/assets/docs/AV).

**Fig. 2:**
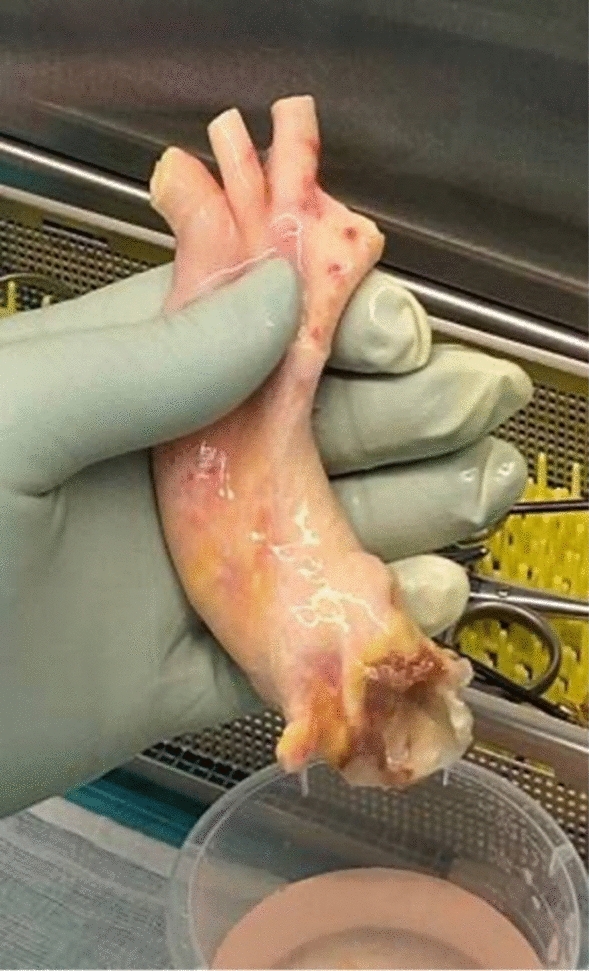
The first homograft harvested for the Odense Homograft Biobank

In 2019, vascular and heart surgeons, together with clinical immunologists at Odense University Hospital (OUH), collaborated to establish Scandinavia’s first cardiovascular homograft biobank (Fig. 2). Here we provide the rationale behind this endeavor, and the process involved in establishing the biobank and the first-year experiences encountered during the first 2 years of using the tissue bank.

The aim of this article is to describe the process of establishing the homograft biobank, the heterogenous population that may benefit from the biobank, the production of homografts, and check the quality quantitatively by in-dept reviewing the results obtained during the first year based on the medical reports as well as qualitatively by very short interviews of the responsible surgeons.

## Methods and material

### Establishment of the Odense cardiovascular homograft biobank

A steering group consisting of doctors and other academic staff from the Department of Cardiothoracic and Vascular surgery T and the Department of Clinical Immunology (DCI) at OUH implemented from October 2019 to March 2021 Scandinavia’s first full cardiovascular homograft biobank. The establishment was based on study trips to other European tissue banks as well as communication and workshops with experts in the field.

A quality management system and a laboratory information system (LIS) for traceability of the vascular homograft was developed by using already existing systems at the blood and tissue transplant service at the DCI, both systems fulfilling the requirement in the EU Directive of Tissues and Cells of 31 March 2004. For labeling and identification of all homograft, the internationally approved standard ISBT128 is used.

Nurses and surgeons were trained in donor selection and in the retrieval procedures for the cardiovascular homograft. A validation of the quality of the graft before and after cryopreservation was performed, including graft strength, sterility, vitality and tensometric strength. Furthermore, validation of the entire workflow including traceability in LIS regarding both donor, graft and recipient. In March 2021, the homograft biobank at OUH was authorized by the Danish Patient Safety Authority to carry out all the above-mentioned procedures including transplantation of homografts. The first homograft harvested is illustrated in Fig. 2.

### Graft harvesting and preparation

Donors are selected according to the criteria in the EU Directive of Tissues and Cells, as assessed by vascular surgeons at OUH. Consent is secured by specially trained intensive care nurses.

The extraction and recovery of blood vessels are performed by transplant vascular surgeons as an immediate extension of the removal of organs for transplantation. Additionally, the removal and recovery of the ascending aorta, aortic valve and pulmonary valve are undertaken by a subspecialized cardiac surgeon.

The quality of the vessels is initially evaluated on a pre-harvesting CT scanning, if there are arteriosclerotic lesions the decision not to harvest this artery can be made preoperatively. During surgery, the arteries are macroscopically evaluated, if arteriosclerotic/degenerative lesions on arteries, varices on veins, or other signs of questionable quality, the vessels are not harvested.

Within 24 h, the grafts are controlled and prepared by a specially trained surgeon. Occasionally, graft material is rejected due to unsatisfying quality, for example minor arteriosclerotic lesions, or minor accidental damages from the harvesting process.

As the maximum storage time before preparation is 24 h, harvesting for the biobank is not performed during weekends or in holidays.

Immediately after recovery, the homograft is placed in individually labeled sterile tissue containers and transported in a refrigerated (4 °C (C)) transport bag to the DCI.

In a clean (AiC) laboratory, the recovered homografts are further processed, including removal of surrounding tissue, measurement and quality assurance procedures. Photography is taken of every single graft and protocolled in the laboratory information system (LIS) for easier selection of the correct graft for the given recipient (see below). Subsequently, the homografts are immersed in a commercial broad spectrum antibiotic liquid and kept at 37 °C overnight (max 24 h), in accordance with the protocols from other European centers. Both pre- and post-antibiotic treatment, tissue samples from all vessels are homogenized using a Gentle MACS dissociator (Milteneyi Biotec) and sent for culture of bacteria.

Following the decontamination, the homografts are rinsed in isotonic NaCl solution, and then cryopreserved in freezing medium consisting of the culture medium RPMI-1640 and 10% dimethyl sulfoxide. The homograft is frozen to − 150 °C using automated temperature-controlled freezing equipment, as depicted in Fig. 1. Following this, it is transferred to the gas phase within a nitrogen container for storage.

### Recipients

Preoperatively: After indication and consent of receipt of a homograft has been given, additional informed consent is obtained to collect and store medical record data in a clinical database. Preoperative blood tests are ordered.

**Fig. 3:**
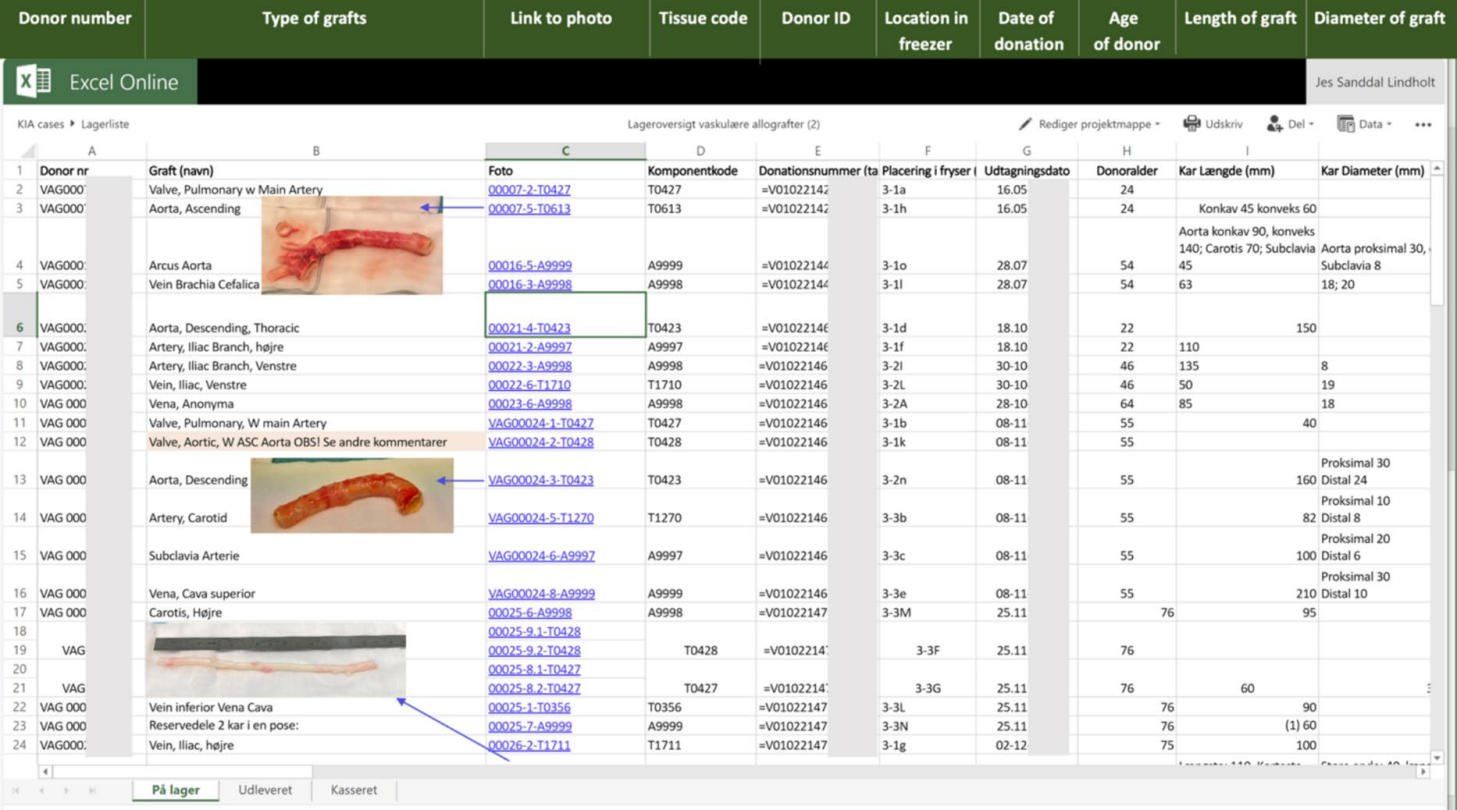
A screenshot of the secured laboratory information system (LIS), where the surgeons select the homografts. (The grey boxes are censored information according to the general data protection rules (GDPR))

Selection of the graft material is performed by obtaining information regarding the donor, the type of graft, its measurements, and a photography of every graft in the bank via a special link on the hospital’s intranet to the laboratory information system (LIS) (Fig. 3).

The choice of the graft material(s) is performed by the surgeon responsible for performing the upcoming surgical procedure. In the elective/subacute settings, where several surgeons are involved, a small informal meeting is often held. We believe this may strengthen the quality of the work and help the team of surgeons to have a clear plan for the operation, and good cooperation around the reconstruction.

As a rule, we only use graft material from one donor in each patient. We prefer this strategy in case immunology play a role in long term degeneration of the graft, or even that there might be cases of acute rejection.

Perioperative: During the procedure, samples are taken for culture and resistance, and recipient vessels are prepared to receive the homograft. Cut tissue is sent for microscopy, etc. description of vitality.

Postoperatively, patients follow the guidelines for each individual procedure in which they are used. The cardiac patients are monitored with echocardiography and CT scans by cardiologists at OUH, while vascular patients are monitored by vascular surgeons including a CT scan after 3 and 12 months, then annually.

### Quality check by interviewing

Surgeons in charge of implanting homografts were informally and semi-structurally interviewed allowing additional experiences expressed regarding the harvesting procedure, the tools for detecting available homografts, their quality and delivery.

### Quality check by reviewing the medical records

Recipients were identified through the laboratory information system (LIS) which records donors and recipients, and their medical records were reviewed by the first author. Twenty-six recipients were identified for this audit.

## Results

### Functioning of the graft harvesting and preparation

Short interviews with the responsible transplantation- and heart surgeons does not indicate special problems with the harvesting process. However, of course they expressed it is relatively time-consuming, meticulous work, and it often has to be performed in afternoon or nighttime.

### Selection of adequate grafts for the patients

During the first year, no responsible transplantation- and heart surgeons have experienced that a suitable graft was not available in the bank, when needed, indicating the laboratory information system (LIS) functions as planned (Fig. 3). We have though noticed that superficial femoral arteries are popular, as their length and diameter make them suitable as components in many different reconstructions.

During the first year we did not experience that the graft delivered was not of macroscopically good enough quality for use. On one occasion, the responsible surgeon found the homograft material too sparse for an aorto-bifemoral bypass, and preferred to solve the problem with harvesting a supplemental short segment of the patients own femoral vein.

Except from this, no special problems regarding the quality of the grafts, or their appropriateness for fitting the selected patients, have been noted in the patient journals.

Comparing list of grafts delivered from the bank, and patient operation journals, we conclude all grafts delivered from the bank was used. This indicates no patients planned for homograft have died on the table, or for other reason did not have the transplantation fulfilled.

### Deliverance from the bank in the elective and acute setting

Graft transplantations were done in the range of 1–2 h from requesting. In weekends and holidays the responsible personal in the biobank may be on call within 30 min.

6 of the operations were performed emergently without certain delay.

According to interview with the responsible surgeons, the deliveries has functioned well, also in the acute setting. Defrosting the graft in the correct way is the time-consuming part of the process which lasts 30 min and thus request timely ordering to avoid waiting time and unnecessary prolonged procedures.

As OUH is a university hospital, a large proportion of patients are transferred from other hospitals for definitive treatment at OUH. The short delay with patient transport can be well utilized for planning the surgery and preparing the selected graft material.

### Deliverance to other hospitals

During the first year two grafts were delivered to other hospitals, one to Haukeland University Hospital, Bergen, Norway, and one to Kolding hospital, Denmark. (The last, but not the first mentioned patient is included in the clinical results part).

Transport of the homografts was done according to the Scandinavian protocol for transporting organs for transplantation (https://www.scandiatransplant.org/).

Delivery to other hospitals will be done if requested, given that graft of good quality with appropriate measurements are available and its production costs covered without profit.

### Clinical results

From the first implantation May 6, 2021, to March 1, 2023, 26 patients have received homografts. Of these, 9 were women and 17 men with mean age and median age of 63.5 and 69 years, ranging from 34 to 91 years. Basic characteristics of the patients are listed in (Table 1).

**Table 1:** Demographics, population characteristics by indication for allograft reconstruction

	All N = 26 (%)	Aortoiliac graft infection N = 10	Mycotic Aneurysm N = 7	Infra Inguinal Graft Infection N = 6	Thoracic Graft Infection N = 3
Male	16 (61.5)	7	7	2	0
Current smoker	9 (34.6)	3	3	1	2
Atrial fibrillation	4 (15.4)	1	1	2	0
Stoke	1 (03.8)	1	0	0	0
AMI	0 (00.0))	0	0	0	0
CABG/PCI	3 (11.5)	1	2	0	0
Valve replacement	0 (0.00)	0	0	0	1
PAD	17 (65.4)	7	5	6	0
COPD	4 (15.4)	4	0	0	0
DM	8 (30.8)	2	4	2	0
Dialysis	0 (0.00)	0	0	0	0
Use of statins	16 (61.5)	8	3	5	0
Use of antiplatelets	17 (65.4)	9	3	5	1
Anticoagulation	7 (26.9)	2	2	3	2
NSAID	3 (11.5)	1	2	0	0
Use of antidiabetics	8 (30.8)	2	4	2	0
Chemotherapy	0 (0.00)	0	0	0	0
Immunosuppressives	2 (07.7)	0	1	1	0
Malnutrition	0 (0.00)	0	0	0	0

Seventeen patients had a reconstruction of the infrarenal aorta and/or iliac arteries: 7 due to mycotic aneurysm, and 10 for central graft infection. Six patients were operated due to infra-inguinal graft infection, and finally 2 had aortic valve for ascending aorta and arch with branches replaced. (Table 2).

**Table 2:** Type of reconstruction and allografts used. All and stratified by indication

Indication	Type of reconstruction	N	Type of allograft	
*Mycotic aneurysm*	*Aortic tube*	5	Desc. aorta	1
			Abd. aorta	1
			Vena Cava Superior	1
			Iliac vein	2
	*Aortobiiliacal bypass*	1	Desc. Aorta + 2 iliac arteries	3
	*Aortobifemoral bypass*	1	VCM + iliac artery + own veins	2
*Aortoiliac graft infection*	*Aortobifemoral bypass*	5	Abd. aorta + 2 a. Fem. Sup	6
			Desc. aorta + 2 iliac arteries	3
			Iliac artery + 2 SFAs	3
			Left and right iliac vein	2
	*Aortobiiliac bypass*	2	Desc. Aorta + 2 iliac arteries	3
			Desc. Aorta + 1 iliac arteries	2
	*Aortounifemoral bypass*	1	V. Cava. Inf + 1 iliac vein	2
	*Iliacofemoral bypass*	2	1 SFA	1
*Peripheral graft infections*	*Fem-fem cross over bypass*	4	1 SFA	4
	*CFA to profund femoral artery*	1	1 SFA	1
	*Fem-pop bypass above knee*	1	1 SFA	1
*Thoracic surgery*	*Aortic valve and asc. aorta*	1	Aortic valve, asc. aorta	2
	*Asc. aorta*	2	Desc. aorta	1
			Desc. Aorta + a. carotis com	2
All		26		43

The different groups were similar according to demographics, though the patients undergoing thoracic surgery were younger than the others, and 70% of patients undergoing surgery for graft infection had their primary prosthesis implanted due to occlusive disease, indicating a more severe peripheral atherosclerosis (PAD) in this group (Table 1). The 26 reconstructions were performed using 45 homografts ranging from 1 up to 3 for each patient consisting of aortic valve, ascending aorta, aortic arch, descending aorta, abdominal aorta, vena cava and iliac veins, as well as the superficial femoral artery, depending upon site of implementation (Table 2).

#### Mortality

Two (7.7%) died within the first 30 days postoperatively, both after aortoiliac reconstruction due to graft infection corresponding to 20% of patients reconstructed for aorto-iliac graft infections. Both deaths were caused by graft failure with severe bleeding. One was diagnosed during re-laparotomy, the other at autopsy. After 90 days the overall mortality rate was 11.5%, but without further graft related deaths.

#### Complications

In all, 7 (26.9%) of the 26 patients were reoperated because of complications.

during the first 30 days and one further (also mentioned above) had a severe graft related bleeding diagnosed at autopsy rising the number of surgical complications to 8 (30.7%). Three (11.5%), were graft related. More specifically, 2 had a re-laparotomy, one for a complex aortic graft related bleeding, and the second for mechanical ileus (Table 3).

**Table 3:** Operative findings and postoperative complications

	All	Mycotic aneurysm	Aortoiliac graft infection	Peripheral graft infection	Thoracic surgery
Number	26	7	10	6	3
*Emergency procedure*	6 (23.1%)	5 (19.2%)	1 (3.8%)	0	0
Operation time (min)		133	215.6	193	462
Blood loss (ml)	1627	1500	2054	279	2675
Blood transfusions (n)	3.34	2	2.6	0.17	8.6
ITU stay (days)	1.33	2.14	0.95	0	1
Hospital stay (days)	11.8	4.6	8.3	1.4	
Length of antibiotics	52.9	48.8	61.3	34	84.7
*Medical complications*					
Stroke	0	0	0	0	0
Myocardial infarction	0	0	0	0	0
Respiratory complication	0	0	0	0	0
Dialysis	0	0	0	0	0
*Surgical complications*					
Reoperation, all	1.75	1pt × 2	2 pt,5 op	2	2
Bleeding	4	1	3	2	1
Reinfection	5	1	3	0	1
Aortointestinal fistula	2	1	1	0	0
Amputation	1	0	0	0	1
*Mortality*					
Overall	15.3	0	17.6%	14.3%	0
Graft-related	7.7%	0	11.7%	0	0
30 days mortality	7.7%	0	11.7%	0	0
90 days mortality	15.3	0	17.6%	14.3%	0

A 34-year-old woman had her homograft aortic valve replaced because of reinfection on day 27 with a freestyle prosthesis.

In the group with infra-inguinal graft infections, two patients had a revision of the groin. One patient, a 42 -year-old man originally treated for A-dissection, had his freestyle prosthesis replaced with homograft due to severe candida infection. He was reoperated due to a non-graft related bleeding, had a sternum VAC, and a leg amputation due to complications with extracorporeal lung assistance (ECLA).

By day 90 two of the 17 (11.7%) patients receiving an aortoiliac homograft reconstruction had developed an aorto-enteric fistula. Both were treated with covered stent.

In all, reinfection occurred in 5 patients, or 19.2% within 90 days postoperatively (the aorto-enteric fistulas were counted as reinfections).

No postoperative medical complications were observered regarding stroke, myocardial infarction, dialysis or intubation for more than three days. Five patients needed attention for respiratory complications, but none of them were reintubated.

### Subgroups

#### Mycotic aneurysms

Seven patients, all men, were operated on suspicion for mycotic aneurysms. They were all alive by day 90. Mean and median age was 69.4 and 73 years respectively, ranging from 48 to 91 years. Three of the patients had contained rupture, another one had a primary aorto-duodenal fistula in relation to the aneurysm, one had a psoas abscess, while one did not show any macroscopical signs of infection at surgery. No agent could be cultured, and further attempt to diagnose the causing agent by PCR were not performed, and the diagnosis of mycotic aneurysm had to be rejected.

Regarding complications, one required reoperation due to mechanical ileus within the first 30 postoperative days, and later developed an aorto-enteric fistula treated with covered stent and prescription of lifelong antibiotics. Consequently, the reinfection rate was 1 out of 7 (14.3%). There were no cases of pseudoaneurysm formation, stenosis or thrombosis in this group.

#### Aortoiliac graft infection

Homografts in this area requested complex combinations of grafts sewed together to form bifurcation prosthesis. Grafts sourced from thoracic and abdominal aorta, common or external iliac arteries, or femoral arteries, vena cava, iliac vein, deep femoral and saphenous veins were used. All combinations are possible.

Ten patients had aortoiliac homograft reconstruction because of suspicion of graft infection according to MAGIC criteria; 3 women and 7 men. Their mean and median age was 64.7 and 69.5 years, ranging from 47 to 75 years. Indications for primary surgery were limb ischemia (N = 7), abdominal aortic aneurysm (N = 2), and iliac aneurysm (N = 1). The number of previous operations in the same vascular segment ranged from 1 to 6 with a median of 2.7.

As mentioned, two patients (20%) died within 30 days postoperatively both by major bleeding. One of them, a 75-year-old man, had his infected aorto-bifemoral bypass replaced by complex homograft consisting of descending thoracic aorta and two superficial femoral arteries. He was reoperated three times due to bleeding, the last time with ligature of infrarenal aorta, removal of the macroscopically necrotic homograft, and revascularized with an axillo-bifemoral bypass. Cultures from the removed homograft was a mixture of facultative anaerobic bacteria. It should be noted that the security culture test of the homograft just prior implantation was negative.

The other case of early death, a 49-year-old woman, had her aorto-bifemoral bypass replaced by complex homograft consisting of vena cava and iliac vein. No cause of bleeding was identified at autopsy, and technical error cannot be ruled out.

By day 90 another patient had been diagnosed with an aorto-enteric fistula and treated with a covered stent and lifelong antibiotics.

#### Peripheral graft infections

Six patients, 4 women and 2 men, had a homograft reconstruction for replacement of infra-inguinal graft infection. Their mean age was 66.7 and 66.5 respectively, ranging from 53 to 80 years. All had primary surgery due to chronic limb ischemia.

Four had an infected femoro-femoral crossover bypass replaced with a homograft, one had replacement of a short femoral graft for the common femoral artery to the deep femoral artery, and one had a replacement of a fem-pop bypass above the knee.

No one died the first 30 days postoperatively, but a severely ill 71-year-old woman died within 90 days from a bleeding gastric ulcer. She was the only patient in this group who suffered respiratory complications. Two had wound revision in the groin within 90 days after homograft implantation but no amputations were performed.

#### Thoracic cardiovascular graft infections

Three cases were identified. One received a homograft consisting of aortic valve and ascending aorta due to an infected mechanical aortic valve but it had to be replaced within 30 days due to reinfection. Two had aortic tube graft infection after surgery for type A dissection and had the ascending aorta and arch replaced. One of these was reoperated the first day postoperatively due to non-graft related bleeding, had a femoral amputation due to ischemia caused by complications to ECLA femoral access and developed sternal infection treated with VAC for some time. He survived.

### Microbiology

Suspected microbiological species and site of graft infection are listed in Table 4. Mainly Staphylococcus aureus and Streptococcus were detected.

**Table 4:** Bacterial findings stratified by type of sampling

Indication for surgery and species	Suspected specific strain	N	Blood-culture	Preop Ulcer or fistula	Perop. prosthesis Tissue or swab	PCR
Aortoiliac graft infection						
* Candida*		2				
	C glabrata	1			x	
	C albicans	1			x	
* Staphylococcus*		5*				
	S aureus	2	x		x	
	S lugdunensis	3		x	x	
	S capitis	1			x	
* Corynenacterium*		2				
	C amycolatum	1#			x	
	C tuberculostaticum	1			x	
* Proprionebacterium*	P acnes	1			x	x
Infrainguinal graft-infection						
* Staphylococcus*		3				
	S aureus	1			x	
	S lugdunensis	2			x	
* Streptococcus*		2				
	Hemolytic streptococcus C/G	2		x		
	Peptostreptococcus	1	x		x	
Mycotic aneurysms		1				
* Candida*		1			x	
	C parapsitosis	1				
* Streptococcus*		2				
	S mitiis	1			x	
	S anginosus	1	x			
* Others*	E coli	1	x		x	
	Bacteroides pyogenes	1	x			
*None*	No agent identified	1^				

*1 mixed infection S aureus/S lugdnunensis

^This pt. had an aortoduodenal fistula in relation to mycotic aneurysm

^#^In combination with S capitis

Regarding the homografts, very few, have been contaminated before antibiotic soaking and refrigerating, and no positive bacterial tests have been noticed on the samples taken at the time of implantation indicating the decontamination procedures works satisfactory.

Regarding the patient who died after several reoperations for bleeding, we suspect infection by facultative anaerobic bacteria may have destroyed the homograft but not due to a preoperatively contaminated homograft as the control culture test of the homograft on the implantation day was negative.

## Discussion

The first Scandinavian homograft bank took 18 months to establish and become authorized by the Danish Patient Safety Authority. We think that this was possible mainly because of scaffold of tissue biobanking was already in place in the Department of Clinical Immunology at Odense University Hospital for other tissues. It included a functioning quality assurance system, existing validated clean rooms, and staff used to work under the requirement of the EU directive of Tissues and Cells. In addition, the collaboration of clinicians, nurses and laboratory technician from different departments of the same hospital worked exceptionally well and was very motivating for all involved. We also believe that the fact that the same clinicians involved in procurement and processing of the homograft are also the end users has a positive effect on the focus on the quality of the homografts.

### Logistics, macroscopic quality of grafts, selection of patients and grafts

The departments experience with kidney transplant, well developed routines in the department for clinical immunology together with the relatively high number of vascular operations, especially aortic surgery with approximately 200 aortic repairs annually, has made the establishment of the homograft biobank possible.

According to our qualitative data the first-year experience of the bank, we conclude it functions satisfactory; there has been harvested enough grafts to fulfill the needs, there has not been any severe problems with the harvesting and preparation processes, the selection of graft for the given patient has in all but one case with sparse material been appropriate, and there have not been incidences where the homografts selected were inappropriate for implantation or been of unsatisfactory quality.

Further, no graft was taken out of the bank without being used, demonstrating competent preoperative decision making and that the patients selected for homograft reconstruction were fit enough to survive the operations.

A weakness by our method is that the qualitative data are mainly based on informal interviews with the surgeons a while after the surgeries causing risk of memory bias. Consequently, we consider to implement a short questionnaire to be filled in for every transplantation, regarding problems with delivery, quality and appropriateness of the graft material as a continuously running quality check of the biobank.

### Clinical results

In this initial cohort of the first 26 patients receiving homograft(s) from Odense Cardiovascular Homograft Biobank, two (7.7%) died within 30 days postoperatively, 6 (26.9%) were reoperated and 5 (19.2%) were reinfected. While these figures may appear high, it is important to note that the sample size is relatively small causing inconclusively very wide confidence intervals. Moreover, these outcomes are not unexpected compared to the literature (see below) due to the severity of the underlying diseases, often advanced age of the patients, and presence of severe comorbidities.

#### Aortoiliac/femoral reconstructions due to prosthesis infection

Among all implanted artificial aortic grafts, 0.3–5% become infected. In situ replacements of the graft (ISR) with either deep femoral veins, a synthetic prosthesis or homograft are preferred, if the patient is considered fit.

Replacement with a new artificial prosthesis carries a high risk of reinfection, which is attempted reduced with rifampicin soaking of the prosthesis and/or silver-coated prosthesis. Alternatively, deep femoral veins can be spliced together to form a “vein-bifurcation prosthesis”, which is more resistant to infection. However, it is a major operation, which is only considered for select cases, with potential complications such as dangerous aneurysm formation or stenosis observed over time.

A meta-analysis of 1377 patients undergoing a homograft for in-situ replacement for aortic graft infection showed a 30-day mortality of 15%, graft related cumulative mortality of 3.6%, degeneration in 5%, rupture in 6%, 25% reoperations, and 3.8% amputees (Table 1) (Chafké et al. 2020).

We observed 2 deaths within 30 days among the 10 patients in this group corresponding to 20% (95 CI 2.5–55.6%) which may appear high, but the very small number of patients indicates that no secure confidence interval can be established and the high number might well be due to chance.

Both deaths were graft related and involved complex homograft configurations, one comprising the superior vena cava and iliac veins, and the other involving the descending aorta and two superficial femoral arteries.

Reoperations occurred in 10% within 30 days and 20% within 90 days, and thus acceptable compared to the mentioned meta-analysis above.

Two other meta-analyses compared surgical treatment methods of 1417 and 1464 patients (O´Connor et al. 2006; Batt et al. 2018). In-situ replacement (ISR) was significantly associated with fewer complications, reinfections and mortality, compared to EAR.

There was no significant difference in the re-infection rate between the different ISR grafts (veins 2%, homografts 9%, Rifampicin-bonded or silver-coated prosthesis (11%), but all showed significantly better results than standard prostheses. Significantly fewer amputations were observed using homografts (3%), rifampicin-soaked grafts (3%), and silver-coated grafts (4%) compared to veins (9%). Standard prostheses exhibited significantly worse outcomes regarding graft occlusion and amputation.

We observed a 10% reinfection rate within 30 days which is in agreement with the above mentioned meta-analyses, but within 90 days as high as 20%, if the aortoenteric fistula is counted as a reinfection. However, as mentioned the 95% CI is very wide.

A recent multicenter study involving 229 implanted aorto(bi)iliac/femoral homografts reported a primary patency of 97% after 5 years, which is comparable to the patency rates observed with the primary implantation of artificial grafts (Batt et al. 2018).

So far no loss of primary patency has happened in our cohort, if reinfections are excluded, and no one has been amputated.

The European guidelines (Chafké et al. 2020) continue to primarily recommend reconstruction using the patient’s own deep femoral veins. Nevertheless, they acknowledge that this intervention is highly extensive and long lasting, making it unsuitable for many fragile patients. In such cases, the guidelines pragmatically recommend the use of cryopreserved homografts, silver-coated grafts, or rifampicin-soaked polyester grafts, and these options are available to more patients, few have the homograft option.

#### Aortoiliac/femoral reconstructions due to prosthesis infection

For mycotic aneurysms, the results were satisfying, in that all 7 patients survived. We haven´t been able to identify for comparison.

#### Aorto-enteric fistula

In two cases out of 17 (11.8%, 95% CI 1.46–36.4%) abdominal aortoiliac reconstructions, an aorto-enteric fistula developed following the implantation of an arterial homograft. The frequency reported in the mentioned meta-analysis was lower, at 3.5% (95% CI 1.19–6.53%), although the sample sizes were small with overlapping confidence interval.

In one of the cases, massive amounts of pus around the graft limbs were observed, yet no microbiological agent was identified. Additionally, antibiotic treatment was accidentally stopped on the 7´th postoperative day. The patient was readmitted on day 31 after a week with melena, and a CT-scan revealed the presence of an aorto-enteric fistula, seemingly originating from a branch of the homograft. However, reinfection cannot be ruled out as a contributing factor. The second case was initially operated for mycotic aneurysm with a psoas abscess.

The cases may have occurred from extensive infection at the time of implementation and/or insufficient postoperative antibiotic treatment. Both patients were treated with an endovascular covered stent, leveraging the favorable outcomes reported in systematic reviews and meta-analysis regarding endovascular treatment of mycotic aneurysms (O´Connor et al. 2006).

#### Aortic valve homograft

Composite aortic valve homografts were predominantly used including the ascending aorta (Fig. 2, Table 1). A metanalysis of 2232 patients with aortic valve endocarditis from 18 studies compared homograft to artificial heart valve for treatment and found comparable survival rates, as well as similar rates of re-endocarditis and re-operation (Williams et al. 2022). A later systematic review and meta-analysis focusing on the treatment of complex aortic valve endocarditis with homograft versus alternative options revealed a 30-day mortality of 17.9% versus 19.4% (RR = 0.91), reinfection 7.5 vs 9.66 (RR = 0.89), and reoperation. 6.9% verss 5.6% (RR = 0.91). While these numbers are relatively small, they suggest a potential risk differential of 10%. Considering these findings, the Society of Thoracic Surgeons recommends homograft utilization for patients with extensive endocarditis with destruction of the aortic ring and should be considered particularly in cases where the risk of reinfection is increased (Williams et al. 2022).

During the first year, three patients had thoracic aorta reconstruction, two of them with aortic valve. One of them had her aortic valve replaced within 30 days due to reinfection. Another had a non-graft related bleeding. As the number of patients is so small, no conclusions can be drawn.

## Conclusion

A fully operational cardiovascular homograft biobank has been successfully established in Denmark, operating around the clock. Although the numbers treated still are small with consequently wide confidence intervals, the initial outcomes demonstrate comparability with those of other centers and alternative methods for reconstructing aortoiliac infections and graft infections, making it a good option for treating infra-inguinal graft infections.

There is a clear indication for continuous monitoring the quality of the grafts and the outcomes which by time will involve larger number of patients, to explore long-term outcomes and identify risk factors for failure. This will enable the optimization of patient selection and homograft utilization.
